# Blockchain Empowered Federated Learning Ecosystem for Securing Consumer IoT Features Analysis

**DOI:** 10.3390/s22186786

**Published:** 2022-09-08

**Authors:** Abdullah Alghamdi, Jiang Zhu, Guocai Yin, Mohammad Shorfuzzaman, Nawal Alsufyani, Sultan Alyami, Sujit Biswas

**Affiliations:** 1 Information Systems Department, College of Computer Science and Information Systems, Najran University, Najran 61441, Saudi Arabia; 2 Graduate School, José Rizal University, Mandaluyong 1650, Philippines; 3 School of Computer Science, North China Institute of Aerospace Engineering, Langfang 065099, China; 4 Department of Computer Science, College of Computers and Information Technology, Taif University, Taif 21944, Saudi Arabia; 5 Computer Science Department, College of Computer Science and Information Systems, Najran University, Najran 61441, Saudi Arabia; 6 Computer Science and Digital Technologies Department, University of East London, London E16 2RD, UK

**Keywords:** federated machine learning, deep learning, blockchain, distributed learning, distributed edge computing, information security, privacy-preserving computing

## Abstract

Resource constraint Consumer Internet of Things (CIoT) is controlled through gateway devices (e.g., smartphones, computers, etc.) that are connected to Mobile Edge Computing (MEC) servers or cloud regulated by a third party. Recently Machine Learning (ML) has been widely used in automation, consumer behavior analysis, device quality upgradation, etc. Typical ML predicts by analyzing customers’ raw data in a centralized system which raises the security and privacy issues such as data leakage, privacy violation, single point of failure, etc. To overcome the problems, Federated Learning (FL) developed an initial solution to ensure services without sharing personal data. In FL, a centralized aggregator collaborates and makes an average for a global model used for the next round of training. However, the centralized aggregator raised the same issues, such as a single point of control leaking the updated model and interrupting the entire process. Additionally, research claims data can be retrieved from model parameters. Beyond that, since the Gateway (GW) device has full access to the raw data, it can also threaten the entire ecosystem. This research contributes a blockchain-controlled, edge intelligence federated learning framework for a distributed learning platform for CIoT. The federated learning platform allows collaborative learning with users’ shared data, and the blockchain network replaces the centralized aggregator and ensures secure participation of gateway devices in the ecosystem. Furthermore, blockchain is trustless, immutable, and anonymous, encouraging CIoT end users to participate. We evaluated the framework and federated learning outcomes using the well-known Stanford Cars dataset. Experimental results prove the effectiveness of the proposed framework.

## 1. Introduction

The Internet of Things (IoT) is an integral part of consumers’ modern lifestyle, which is constantly increasing. According to statistics, the number is estimated to reach 14 billion by 2025, and the market will reach USD 142 billion by 2026. Data from these devices is expected to reach 73.1 ZB (zettabytes) by 2025 [1,2,3]. Managing so much data and maintaining every security and privacy of users is very challenging. However, nearly 94% of retailers agree that the benefits of implementing IoT outweigh the risks [2].

CIoT uses smart systems coupled with several advanced technologies such as wireless communication, cloud computing, edge computing, big data analytics, artificial intelligence (AI), etc. Such intelligent systems generate large amounts of data that can be a significant asset in predicting upcoming challenges. Additionally, they can help improve existing systems through smart big data analytics and machine learning. However, both technologies analyze stacked data from a centralized system, raising security and privacy concerns; leading technologists came up with federated learning technologies. Local nodes in FL train a model locally using self-training data and share the learning results as model parameters to the aggregator instead of sharing the raw data. It overcomes the challenges of sharing local data on a centralized server and enables learning through decentralized analysis of locally generated data. At the end of every round of training, all FL servers share their model to a centralized aggregator, which makes them average and creates a global model. Consequently, the global model is used for the next round of training.

However, many studies have recently been published on data security and privacy vulnerabilities in FL technology [4]. For example, authors [5,6] claim intruders can retrieve raw data (i.e., training data) from gradients processed in a centralized aggregator. Moreover, many intermediary devices (e.g., smartphones, Edge Server (ES), etc.) between the data source and the aggregator play the role of a gateway in the CIoT system. These gateways can be another leakage point of data. Third-party MEC servers and smartphone applications as a gateway can leak CIoT raw data by abusing its permission system [7]. Although FL was introduced to stop data leakage, gateways in an ecosystem can leak raw data, and FL’s centralized aggregation policy can lead to model poisoning attacks [8].

Many recent contributions suggested a blockchain-based decentralized model aggregation platform instead of a centralized aggregator. For example, ref. [9] proposed a blockchain-based FL platform for learning IoT features. Similarly, BC-based FL is proposed in [9] to prevent industrial data leakage. It is a fact that BC can overcome the centralized averaging issues. However, the CIoT platform is quite different than any typical IoT. Whether CIoT is a resource constraint and data is carried over the gateway is another IoT but more resource intensive. Data leakage prevention from any point, including the gateway, is also essential to ensure security and privacy in an ecosystem. Therefore, it is crucial to design a non-interactive and privacy-preserving FL scheme to create a secure ecosystem that can prevent privacy leakage from every connection from data acquisition to final prediction, including local gradients, aggregators, etc.

This paper contributes a blockchain-based federated learning framework for a consumer IoT ecosystem that ensures consumers’ privacy-preserving and facilitates future prediction for system advancement. The proposed FL system builds the machine learning model to help CIoT manufacturers improve their service quality and optimize their functionalities. Furthermore, blockchain opens a secure access channel for smart system users and intelligent systems controllers or stack holders by controlling the authentication of every component, such as gateway, ES, etc. Moreover, a BC-based system prevents malicious model updates by ensuring that all model updates are held accountable. The core contribution of this research includes:

- A blockchain-based privacy-preserving FL learning framework for IoT ecosystem that leads the future prediction from the features of CIoT data.
- A secure decentralized secured model aggregator instead of centralized that overcomes the single point of failure.
- A secure access channel protects against man-in-middle attack issues.
- A testbed implementation using a popular public Stanford Cars dataset, and the evaluation result proves the proposal’s effectiveness.

The rest of the sections of this article elaborates on the details of implementations. More specifically, Section 2 presents related recent contributions in blockchain-integrated FL. Proposed architecture details are depicted in Section 3. Section 4 presents technical details of core contributions including Security discussion. Testbed setting, implementation parameters, and results analysis are presented in Section 5. Finally, Section 6 concludes the overall contribution.

## 2. Related Work

Blockchain and FL techniques are widely considered for the security and privacy of data independently where focused on distributed execution. Both private blockchain and federated learning emerged around 2016. Google introduced federated machine learning technology for decentralized training, replacing typical centralized training strategies with user data protection in mind [10]. They suggested combining each local model on a centralized server to create a global model with their average and use it for the next training step. From a security standpoint, this centralized coordination of the FL is recognized as one of the downsides in [11]. The prime objective of FL is data security and privacy, which was investigated in [12], where they considered the model’s safety at updating the aggregator. In [13], authors contributed artificial intelligence for ensuring efficient and privacy-enhanced federated learning (PEFL) for IIoT. They solved various industrial challenging problems in Industry 4.0. Likewise, privacy issues at sharing model updates in FL were investigated in [14] contributing to a sketching-based FL. Protecting the user-level privacy leakage in FL against attack from a malicious server has been studied in [15] where they considered A generative adversarial network.

Business blockchain introduced for eliminating centralization problem in a private network [16]. Initially, blockchain is mainly considered for secure distributed ledger and FL for distributed training in the ML domain. For example, Deep-Chain proposed for collaborative learning in [17]. They provided blockchain to force the participants to behave correctly and focused on ensuring audibility for the whole training process targeting data privacy for each participant. However, how updated models will be uploaded is not clear. Similarly, the authors [11] propose a blockchain-based decentralized approach to local gradient sharing where blockchain stores models. The challenge is how a block with weight limitations can adopt such a heavy model. Blockchain was also considered for improving the training process in many articles [18,19]. The smart contract-based data model provenance registry framework was proposed to ensure accountability and fair distribution of user data in [18]. In addition, they have used a weighted fair data sampler algorithm to increase the training data’s fairness to improve the training quality. Authors [19] contributed to a blockchain-based framework to influence high-quality data owners in FL by providing a reward allocation mechanism. To speed up the training process and minimize the computation cost, the blockchain-based secure model migration technique was introduced in [20]. Technically, the blockchain-focused reputation mechanism produces high-quality model aggregation transparently. Like other contributions, BC plays a reward and credit point calculation role.

Many of them have raised these general issues of FL, its limitations for different applications, and suggested bridging FL and blockchain. Combining these two technologies to preserve decentralized and heavy exchange models for consensus processes is more challenging. Moreover, saving extensive data in the block is another challenging task. The article [21] tried to touch on these (i.e., storage and consensus) issues and proposed a committee consensus for collaborative decisions in the blockchain network. They recommended partial nodes for storing abandoned historical blocks to release the storage space, and a committee for consensus helps to reduce the chances of malicious attacks. In reality, storing models in the active chain and the continuing consensus is more challenging and raises scalability and bandwidth issues.

However, the ultimate goal of most of these articles was to overcome the problem of centralization at model aggregation using blockchain technology. Contrarily, the role of blockchain and how it can ensure the most technologically advanced benefits, such as access control, the overall security of the ecosystem, man-in-the-middle attack, consent, ledger maintenance, etc., has not yet been precisely defined. Although blockchain for protecting against man-in-the-middle (MIM) attack in GW has been studied in [22,23]. They focused on how differently Man-In-the-Middle (MIM) attacks can happen in domains other than ML. To the best of our knowledge, a MIM attack is not considered in a blockchain-controlled ML-based IoT ecosystem.

This research focuses on an FL learning compatible BC network that introduces a customized block structure and multi-chain. The Table 1 presents the summary of the differences between these contributions with contemporary contributions. Block stores model file reference instead of the original file, and organization-specific off-chain storage holds the original file. While blocks carry only a model file pointer instead of the original file, it resolves the overburdened issue of blocks and enhances security. Furthermore, a certificate authority-controlled security architecture ensures the security and privacy of the overall ecosystem, including the protection of MIM.

## 3. System Architecture

**Figure 1:**
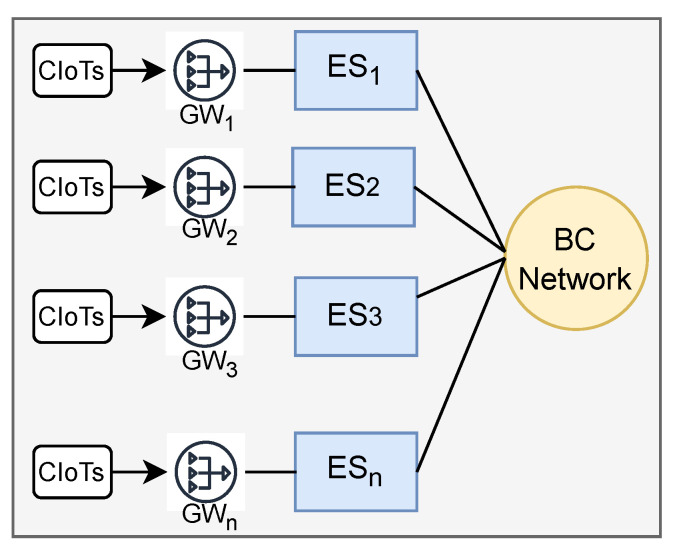
Basic connectivity of components in the proposed ecosystem.

The basic architecture of the ecosystem has been presented in Figure 1, where real-life devices are introduced as CIoT. These resource constraints CIoT are connected with ES via GW. Finally, the blockchain Network (BCN) links to each ES and controls the overall access policy of the ecosystem. System architecture comprises system overview, features extraction of source data, task offloading, and learning process. The system overview briefly summarizes the overall system components’ connectivity and execution. Feature extraction presents data origination to feature extraction for learning features, offloading detailing run-time obstacles or errors. Overall, FL execution processing is described in the learning process.

### 3.1. System Overview

**Figure 2:**
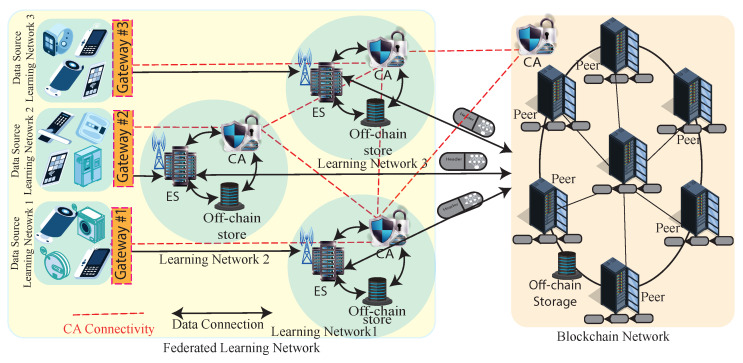
Blockchain-based federated learning framework for CIoT ecosystem.

An overall ecosystem with details of the connection of components is presented in Figure 2. It shows how a group of IoT (e.g., wearables, home appliances, Internet of Medical Things (IoMT), etc.) is controlled through a consumer gateway and stores the raw data. Since most CIoT’s resource-constrained, users access, control, and monitor them through a gateway that belongs to the user’s personal property, such as a laptop, personal system, advanced network router, etc. It is assumed that any cloud services integrated with CIoT services are also agreed to exchange CIoT data with ES that acts as a learning node (e.g., machine learning compatible server). Due to security reasons, GW executes Convolutional Nural Network (CNN) layers of an ML algorithm and extracts the features; consequently, connected layers are executed in LN. LN is authorized by the BC network and collaboratively operates the machine learning process. LN node updated model is aggregated in the blockchain network, a separate distributed network. BC network is also responsible for maintaining authentication of LN [24], GW, and CIoT.

#### 3.1.1. Federated Learning Network

The FL network consists of independent learning networks. Each learning network represents an independent organization that leads by a Learning Node (LN). The Edge Server (ES) of an MEC network functions as an LN also. Each network shown in the Figure 2 has an Edge Server (ES), off-chain storage, and a Certificate Authority (CA). CA and off-chain storage have been added for secure collaboration between networks and storing physical model files.

- **Learning Node:** A learning node represents the central coordinator of an independent learning network and an MEC network. The ES in the MEC network controls the remote devices through a gateway device. It is assumed that each GW has at least one LN. LN learns from connected layers and trains a local model where its integrated GPU executes ML algorithms and CPU functions as an ES. LNs collaborate through BCN and form a learning consortium where the CA confirms their authentication.
- **Certificate Authority (CA):** The Certification Authority is responsible for issuing unique certificates for each component of an organization (e.g., LN, GW, edge component, user, remote device, etc.). Each organization belongs to a CA that collaborates with others to exchange their certificates. In addition, CA is responsible for ensuring the enrollment of new peers and LNs in the BCFL network. Finally, the BC peers justify the issuer’s credentials before approving the transaction.
- **Off-chain Storage:** Each network has off-chain storage that stores model update files. LN can carry off-chain data in real life. Usually, off-chain data is any nontransnational data that is too large to be stored in the blockchain efficiently and requires the ability to be changed or deleted [25]. FL-compatible blockchain is not like a conventional public blockchain network (e.g., Bitcoin, Ethereum, etc.) considering transaction nature. The model with the update parameters generated after each round is too heavy to fit the block. Instead, the off-chain storage model carries the file and issues a file reference. The reference is attached to a block as a file pointer and is executed as a regular blockchain transaction.

#### 3.1.2. Blockchain Network

The overall network architecture comprises a federated learning network where a blockchain network functions as a distributed aggregator and access controller. The blockchain network maintains permissioned blockchain in principles where CA controls the permissions [1,26]. Different consumer CIoTs are connected to BCN through their GWs. Blockchain network-centric collaborative learning networks form a learning consortium where the BC network provides an autonomous service for collaborative learning outcomes. The BC network consists of peers (*P*) and a Certificate Authority (CA). Peers are interconnected and form a Peer-to-Peer (P2P) network. In this P2P network, peers bridge independent ES and open the scope of collaboration that ultimately forms a consortium. The core component of the network *P* handles the consensus process and stores global model references in the ledger. *P* adds consensus participating blocks to its local ledger at the end of a consensus session. CA^BC^ in the blockchain network is also interconnected with the CA^LN^ of the local learning network. Before any device integration to the network CA^BC^, create unique credentials for the device and exchange them with all CA^LN^. They create a compound key for their mutual agreement (details in Section 4). Devices or components use their keys for any transactions. As shown in Figure 2, CAs’ connectivity (’red’ line) forms an independent network. It also maintains a 51% consensus for adding or disconnecting any devices. So, if somehow any of the CA is out of order, the system can issue the required credentials.

### 3.2. Features Extraction

Various terminal devices and communication nodes (i.e., CIoT, cameras, GPS devices, etc.) periodically perceive home appliances’ parameters. Collected data forwarded to gateway devices via network layer after analog-to-digital conversion. The home user starts training the model using collected data. The Convolution Neural Network (CNN) is the feature extractor to extract features from the original data in the gateway. Consequently, fully connected layers are uploaded to the ES server. For experimental purposes, we have used a generic computer for adopting the partitioned deep learning model training approach [27]. In real-life scenarios, the powerful latest smartphone can be used as a gateway for feature extraction and partial training. Ensuring gateway security, we have used a CA-issued certificate for secure connectivity (details in Section 4.4).

### 3.3. Local Training

It is assumed that each ES agrees to be governed by the BC access control policy through a smart contract. CA initiates the required credentials for authentication and secured access. Blockchain-controlled ES joins the local training process by maintaining the following steps.

- Initialization: any gateway device under the full control of a home user is decided to join the FL training session. Interested customers download the initial model from the blockchain network.
- Training: Gateway offloads the privacy-preserving features to the ES that helps train the fully connected layers. The edge server completes the local training rounds and produces loss and a local model. The local model is stored in off-chain storage, which Inter-Planetary File System maintains (IPFS) [1]. The details of model offloading and local training procedures have been discussed in the next sections.
- Block Creation: A block creates with training loss of the last epoch, a hash of the model, and a hash of the physical location of the model. Consequently, a newly generated block is transmitted to the gateway, which the consumer controls.

### 3.4. Global Model Selection

Consumers sign the block with their private keys and upload it to the blockchain through their gateway. Blockchain nodes create a leader panel for every consensus session randomly. The consensus session is based on time (e.g., one minute), defined by the blockchain network controller (i.e., IoT manufacturer). The top leader collects all blocks coming to the network. If the top leader fails, BCN autonomously chooses the next leader from the panel. Here, it is mentioned that all members are concerned about participating blocks. The leader is responsible for averaging the models in the session, creating a new block, and forwarding it to everyone with all blocks hash as metadata. Consensus member verifies the block hash and signs on it by gossiping protocol. Finally, the leader ensures two-thirds of the members sign on it and form a new block that holds a global model. Consequently, it is transmitted to every member and is a gateway to starting the next round of training.

## 4. Technical Details

This section summarizes the core technical implementation theory in ES and blockchain networks.

### 4.1. Training Management

The ultimate goal of FL is robust learning of local users and aggregators without data sharing from data owners. For leveraging the learning process user runs the local model ($M^{l}$) using his data and forms a global model ($M^{g}$) with the collaboration of other users. We used blockchain as a global model initiator, which transmits the parameters $M^{g}$ of the global FL model to GW (i.e., users), where the local training executions are performed on behalf of users. $M^{g}$ creates every round of training by averaging the local models, which is presented in Equation (1).

(1) $$M_{i}^{G}=\frac{1}{D}\sum_{i=1}^{n}M_{i}^{l}$$

For the learning process, we design a generic FL model where a user *i* collects and process a input a matrix $X_{i}=\left[x_{i1},x_{i2},\cdots x_{id_{i}}\right]$ of input data where $x_{id}$ is an input vector of FL algorithm. Let consider $Y_{id}$ is the output of $X_{id}$ and output data vector for training using the FL algorithm of a local user $GW_{i}$ is $y_{i}=\left[y_{i1},y_{i2},\cdots y_{id_{i}}\right]$. A vector $w_{i}$ determines the parameters of the local FL model ($M^{l}$). For example, $x_{id}^{T}w_{i}$ represents the predicted output in a linear regression algorithm, where $w_{i}$ denotes the weight vector that determines the performance of the linear regression learning algorithm. Aiming for training loss minimization, local user *i* seeks to find the optimal learning model parameters $w_{i}$. The training process of an FL algorithm is performed by

(2) $$M^{G}=\frac{1}{D}\sum_{i=1}^{u}\sum_{d=1}^{d_{i}}f\left(w_{i},x_{i,d},y_{i,d}\right)$$

where $D=\sum_{i=1}^{n}D_{i}$ is the sum of training data of all participating users where $\frac{1}{D}$ used for averaging, $M^{G}$ is the global model, and $f(w_{i},x_{i,d},y_{i,d})$ is the loss function. As we know, the FL algorithms’ performance varies on the loss function. Moreover, it varies for different learning tasks. For example, for learning task prediction, the loss function captures the prediction accuracy of FL. Contrary, the loss function captures the classification accuracy for a classification learning task. Therefore, the algorithm-wise FL loss function can be defined [28].

The performance of FL algorithms depends on both $M^{G}$ and $M^{l}$ more clearly after initialization. The update of each user *i*’s $M^{l}$’s weight parameter $w_{i}$ depends on $M^{G}$ while the update of the global model $M^{G}$ also depends on all of the users’ local models. The update of the local FL model $w_{i}$ depends on the learning algorithm and optimization algorithm. We have used the Stochastic Gradient Descent (SGD) algorithm to update the local FL model.

### 4.2. Computational Convergence and Complexity

As different third parties operate edge servers, deploying them decentralized to ensure the same level of security, transparency, and privacy preservation is hard and complex. However, blockchain technology can essentially overcome the shortcomings. In addition, edge computing can provide necessary local computing capabilities, which enable federated learning facilities, and also allow computation tasks of blockchain systems, e.g., smart contract execution and consensus procedure. Therefore, the convergence of blockchain and edge computing paradigms can enable security, privacy, and scalability. An edge server’s complexity is handling massive data volume coupled with advanced networking technology (e.g., SDN, NFV, etc.) [29]. In addition to local model training and handling the offloading task. Details of the offloading computation have been discussed in Section 4.3).

### 4.3. Offloading

It is assumed that *n* number of a gateway controls *n* group of CIoT, which can be denoted as $\left\{GW_{1},GW_{2},\cdots GW_{N}\right\}$ where *m* number of CIoT is active under a gateway which can be expressed as $D_{i}^{1},D_{2}^{i},D_{3}^{i}\cdots D_{m}^{i}\in GW_{i}$. *n* number of gateway connected with *n* number of Edge nodes $E^{n}$ which means $GW↔E^{n}$ where $\{E_{1},E_{2},\cdots E_{n}\}\in ES$ are blockchain controlled. For training, GW collects raw data from devices and continues feature extraction by the CNN process. Simultaneously, it takes the offloading decision based on the computation capacity of the gateway. If the local gateway can do all computation, it continues and sends the model to BCN. Otherwise, it offloads the connected layers to the blockchain-controlled ES. It is assumed the offloading task $T_{i}$ requires $C_{i}$ CPU cycles and α(α∈[0,1]) denotes the offloading decision where α=1 means offloading requires and α=0 express offloading does not require.

#### 4.3.1. Offloading Rate ($R^{T}$)

The model offloading from the gateway to ES is a calculation of the uplink rate. We have considered Shannon’s theorem [30] to rate offloading the tasks to ES.

(3) $$R^{T_{i}}=B\log_{2}\left(1+\frac{C_{i}^{GW_{i}}}{L^{GW_{i}↔ES_{i}}}\right)$$

where *B* is the channel bandwidth, $C_{i}$ is the computation capacity of the GW_i_, and latency between GW_i_ to ES_i_ is denoted by $L^{GW_{i}↔ES_{i}}$.

#### 4.3.2. Computation

Task computation is the summation of local computation in GW and remote computation for offloading in FS. Based on the offloading decision, the local remaining computation task will be $\left(1-\alpha_{i}\right)C_{i}$. Then, the local computation delay can be

(4) $$\delta_{i}^{local}=\frac{\left(1-\alpha_{i}\right)C_{i}}{\lambda_{i}^{local}}$$

where $\lambda_{i}^{local}$ denotes the CPU cycle frequency, in cycles/s.

For remote computation in ES, the overall computation is performed in two ways such as data transmission and task computation. Data transmission delay depends on the transmission rate. Data transmission delay is calculated by Equation (5).

(5) $$\delta_{i,tran}^{ES}=\frac{\alpha_{i}D_{i}}{R^{T_{i}}}$$

where $D_{i}$ denotes the offloaded data size.

One ES can be connected to multiple GWs and is required to process their data in parallel. Therefore, the computation resources of ES_i_, $C^{ES_{i}}$ will be reasonable divided. So, the weighted average division of resources based on the offloading tasks is calculated as:

(6) $$C_{i}^{ES}=\phi_{i}\lambda^{ES_{i}}\frac{\alpha_{i}C_{i}}{\sum_{i=1}^{m}\alpha_{i}C_{i}}$$

where $\phi_{i}$ denotes the proportion of total resources occupied by the task offloading process. Rest $\left(1-\phi_{i}\right)$ processed in the blockchain. Finally, Equation (7) calculates the computation delay in ES.

(7) $$\delta_{i,com}^{ES}=\frac{\alpha_{i}C_{i}}{\lambda_{i}^{ES}}$$

Therefore the overall delay for task offloading in ES can be calculated by Equation (8)

(8) $$\delta_{i}^{ES}=\delta_{i,tran}^{ES}+\delta_{i,com}^{ES}$$

### 4.4. Blockchain for Access Control

Blockchain is widely used to enforce security, privacy, and access control, besides ledger management [31]. In the blockchain, members’ access rights are verified and controlled through smart contracts. We have proposed a channel-based access control policy in addition to smart contracts that protect against any unauthorized access to GW. Specifying a channel per GW access is primarily controlled with a *compound key*, created using channel members (i.e., user, GW, ES) public-private keys. A channel $C_{i}\in C$ is created per GW through a policy transaction approved by blockchain consensus, which is stored as *config* block in CA^BC^. The user owns the channel, while GW and ES are members Algorithm 1 shows the secret compound key (sck) generation process.

**Algorithm 1:** Compound Key Generation Process** Input**: $\left(C_{i}^{id},U_{i}^{id}\right)$** Output**: (Compound.key(sck))**1**  Initialize $\mu^{U_{i}}\left[\right]$   a list
**2** $sk_{\varepsilon_{nc}}^{U_{i}},pk^{U_{i}}\leftarrow \mathrm{cryptogen}\left(\varepsilon_{nc}\left(\right)\right)$// Generates unique key for users$U_{i}$**3**  $pk^{U_{i}}\to \forall_{\mu_{i}\in \mu^{U_{i}}\left[\right]}$// All public keys are grouped**4** $Resp\left[\right],\;pk^{U_{i}}\left[\right]\leftarrow \mathrm{Response}\left(\mu_{i}^{U_{i}}\right)$// Group keys return with approval**5**  **if***all Resp[] are valid***then****6**  | $\mathrm{sck}\leftarrow \mathrm{Compound}.\mathrm{key}(pk^{U_{i}},pk^{\mu_{x}}\left[\right],sk_{\varepsilon_{nc}}^{U_{i}})$
// Compound key generates
**7**  **end****8**  Return sck

In line 2, CA^BC^ generates key pairs by cryptogen() (which is a common tool used in CA). Line 3–4 sends the public key of User $U_{i}$ to every selected member (μ), which means the user is authorizing them to access data. Members return their public key in response, and a response value is recorded as a list. Finally, if all responses are accepted, then a compound key is generated, a combination of all members’ public keys and secret keys of $U_{i}$. It is then shared with all channel members. It is important to note that compound() generates an irreversible hash value as the compound key.

#### 4.4.1. Distributed Ledger (DLT)

**Figure 3:**
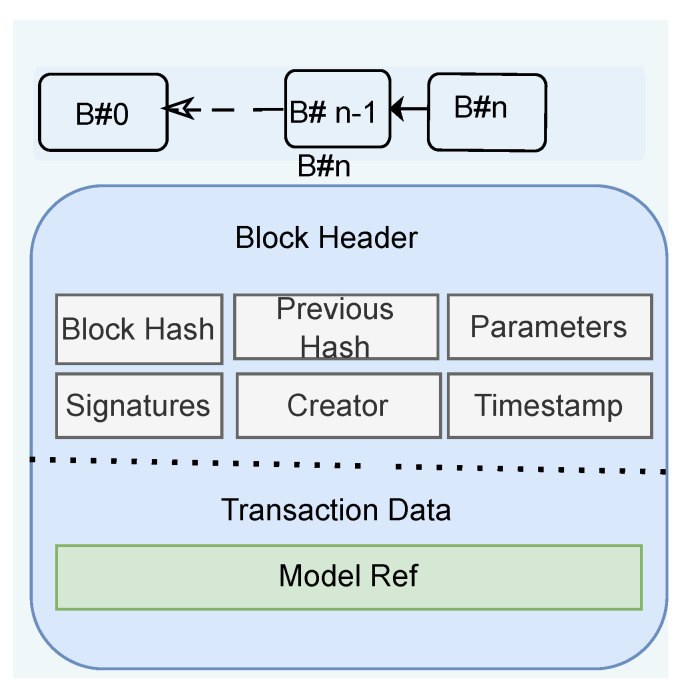
Customized structure of Block used in blockchain.

FL compatible block is very different than typical blockchain (i.e., Bitcoin, Ethereum, etc.). Instead of any numerical data, it is required to store the model file as data that is heavy in size in comparison to crypto-BC. As shown in Figure 3, a block is mainly formed with a block header and transaction data in the body. Block header contains block hash generated from a hash of a model file, timestamp, and file pointer of a model that indicates the physical location of the model and parameters. Every new model generated in the consensus session, known as a global model, is also stored in a new block, maintaining the same structure but with the consent of consensus participants. A newly created block will be linked with the last generated block, so the overall process sequentially creates a model chain.

#### 4.4.2. Consensus Mechanism for Global Model

The main objective of consensus is to generate a global with the consent of a maximum number of members. The global model is generated by averaging every local model on a current consensus session. Every LN creates a new block at the end of every training iteration and forwards it to BCN. BCN randomly initiates a consensus leaders panel using gossip protocol [32]. The top leader from the panel starts a consensus session for a particular time and waits for the local models (as a block). Member peers forward their model to the leader. The leader verifies the credentials (i.e., sign, keys, etc.), calculates averages, and adds to a new block. The global model file is stored in off-chain storage in IPFS, and a hash of the model location pointer is added in a new block. Consequently, the leader invites members to sign on to the new block. Peers justify the block hashes used for averaging, the leaders’ signature, etc., and sign the block. Then, leaders wait for the consent of at least 51% of the total participants. While two-thirds of members sign on it, the block is added to the ledger. Consequently, the block is shared with every peer for upgrading their ledger. The leader also forwards the global model to the LNs for the next round of training.

### 4.5. Security Discussion

The malicious learning node and peer can influence the learning outcome in two ways: an external node being a network member or the adversarial role of a member node. An LN can poison the learning parameters and influence the global model. A malicious node must be approved by CA being a member of the system, and CA can only allow membership if 51% CA agrees on it. In addition, the peer node addition request is also approved by the peer network as per the smart contract agreement. As a member of the ecosystem, intruders can send a malicious model to influence the outcome. However, practically, LN updates the model depending on a consensus outcome where every participant votes by justifying every block sent from LN, verifies signatures, etc. Malicious nodes can be successful if they are 51% in numbers and does several operations within a particular consensus session. For example, they have to retrieve maximum private keys, control off-chain storage, and break up the smart contracts installed in every peer with a consensus, which is almost impossible. Usually, a consensus session is a tiny amount of time; in our experiment, we used 60sec to 180sec. In real-life scenarios, it will be shorter. As the LN node stores data in its off-chain storage, and the LN itself owns the data. Therefore, model sharing is restricted at consensus sessions in read-only mode. Moreover, smart contract limits access within consensus purpose.

## 5. Evaluations and Discussion

**Figure 4:**
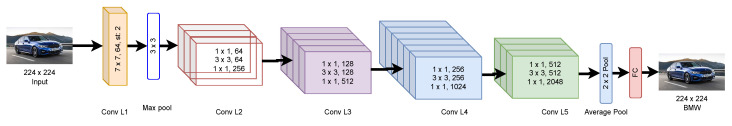
ResNet50 CNN network architectures.

To evaluate the effectiveness of our proposed blockchain-based FL framework, we perform it on a Stanford Cars Dataset [33] that contains 16,185 images of 196 classes of cars. The data is split into 8,144 training images and 8,041 test images. To understand the proper method impact, we have equally distributed data from every class and formed a customized dataset for every organization. The same customized dataset is used in typical FL without blockchain for benchmarking. Furthermore, the experiment extends using the same experimental setup with the entire dataset. Our designed CNN network contains a hidden layer for feature extraction and fully connected layers for classification (shown in Figure 4). Dimension is reduced for output by deploying the max-pooling layer. Therefore, max-pooling layers accelerate the learning speed of the neural network. After every CNN layer normalization is used, that enables the computation of sensitivity to determine the amount of noise to add, speeds up the learning rate and regularizes gradients from distraction to outliers.

### 5.1. Testbed Settings

We train our models with the PyTorch library, SGD optimizer, and a learning rate of 0.001. Each local organization (learning node) performs the classic image classification pre-trained ResNet50 model. Each learning node is used on NVIDIA GeForce RTX 2080 GPU. Initially, four private servers with four GPUs, which means 16 GPUs are used for multiple local training environments. For experiment purposes, every GPU runs an independent learning node. Simultaneously, the CPU of a private server performs as a blockchain peer. The blockchain network consists of six peers running on four remote servers, each configured with Intel Xeon E7 v3/, Core(TM) i7-5960X CPU @ 3.00 GHz 8 cores and 125 GB RAM. The blockchain network and consensus process are simulated in Python 3.8.

### 5.2. Result and Discussion

This section presents overall learning outcomes related to the proposed blockchain-based federated learning framework. We have used typical FL approaches as a baseline for the bench-marking parameter. Since federated learning is still not mature enough, experiments using the same dataset and testbed parameters are also unavailable to compare. Table 2 presents a comparative analysis of training performance with state-of-art where the contributions are evaluated using a different dataset.

Table 3 summarizes the time consumed at different process execution stages in the ecosystem. It shows almost every execution time limit within Millisecond (ms) level except training and aggregation. Local training time is 4–8 min (5280 ms on average) at every LN, which can vary depending on the input data’s complexity and size. Similarly, averaging also takes more time due to averaging of every local model and consensus process as we have fixed different consensus times (3 5 min) for a different level of the experiment, the execution is completed within the consensus time limit otherwise.

**Figure 5:**
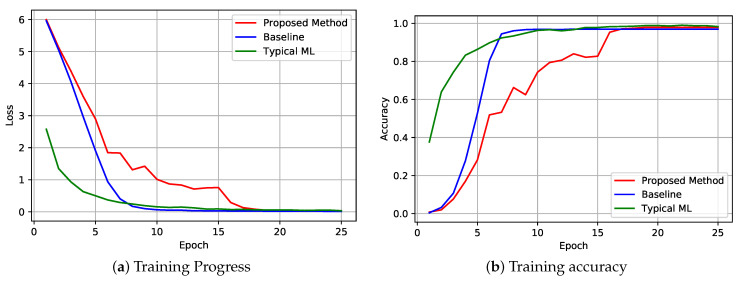
Federated Learning Outcomes in the proposed framework.

We have employed 100 epochs on 8144 training images with 64 batch sizes to train and validation the proposed model. The validation dataset and training dataset are created from training images where 20% images are used for the validation process and the rest of the images for training. We split training images into six learning nodes equally based on their classes. Every LN continues training simultaneously without overlapping training images. The Figure 5 illustrates the training outcome in detail. Training progress has been depicted in Figure 5a where the learning rate was 0.01 and batch sizes 64. As shown in Figure 5a, training loss decreases steadily in our proposed method compared to baseline and typical ML, but it reaches the convergence point at almost the same time.

Training accuracy presented in Figure 5b. It shows the learning accuracy of the object detection model in our proposed system, which is compared with typical federated learning and stand-alone typical machine learning approaches. The simulation result shows that the proposed framework converges with typical approaches almost at the same time.

**Figure 6:**
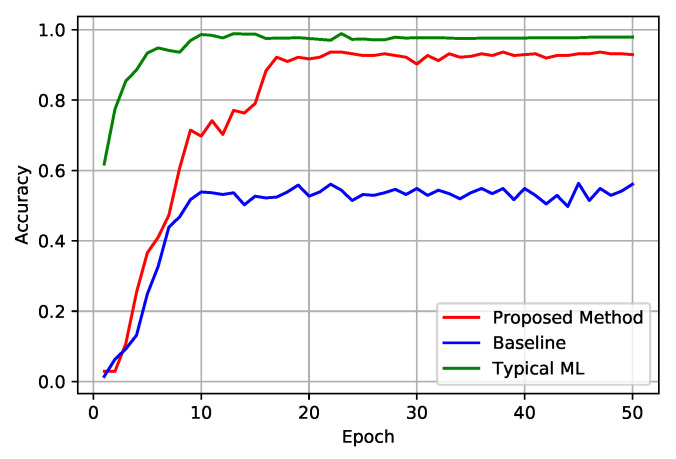
Validation Accuracy.

Figure 6 presents how our proposed system can classify the images compared to typical FL and stand-alone ML approaches. It illustrates that our proposed scheme can recognize the images 92% cases, which is 35% better than the baseline approach. Although stand-alone methods perform better than our proposed system, it is not significantly too high. In terms of security, the proposed methods open up more opportunities than typical ML.

Moreover, the ultimate goal of this experiment is to improve FL in terms of users’ data security and privacy. Therefore, the overall performance of our proposed system is relatively better than traditional FL approaches, which proves the efficiency of the proposed system framework.

**Figure 7:**
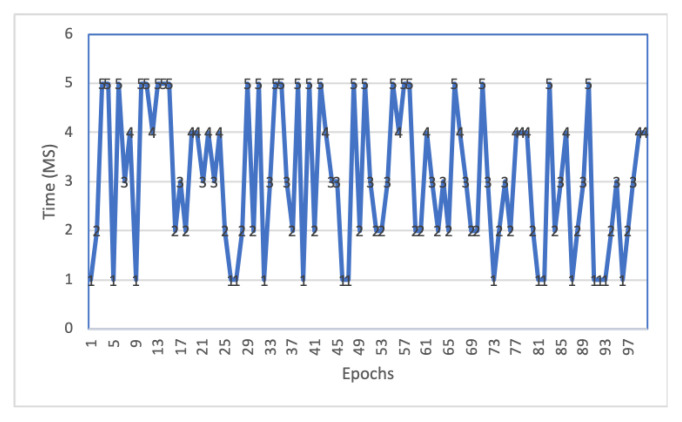
Block Creation time.

The performance of the blockchain network is evaluated in Figure 7. It shows that every consensus round creates a block for the global model. The figure illustrates that block creation times vary between 1–5 milliseconds. Block creation at a millisecond level is satisfactory network performance and is almost consistent. We have set up a 3–5 min consensus session to investigate the BC network’s performance with a number of participation of LN in consensus, such as participation of every LN node, maximum nodes, etc. We have investigated that a global model can be generated with the participation of six learning nodes within 3–5 min. Though the time can vary depending on the input size, batch, bandwidth, etc.

## 6. Conclusions

This article introduced a blockchain-based novel federated machine learning framework for consumer IoT data analysis. We have considered every security challenge in an ecosystem, such as data leakage scopes in the gateway. We recommend blockchain for complete access control of the ecosystem besides distributed model aggregators. Furthermore, using a consensus process for global model creation extends the security in model sharing and accelerates the learning process. Extensive assessment findings from real-world datasets illustrate that the best model selection technique based on consensus enhanced safety and significant output differences from traditional FL approaches. A blockchain protocol-independent framework for federated machine learning can be the future challenge of this research.

## Figures and Tables

**Table 1:** Summary of related contributions.

Reference	Contribution	Distributed Training	Access Control	Ecosystem’s Security
[17]	Secure model sharing	✓	X	X
[20]	Acceleration of training	✓	X	X
[12]	provenance of model	✓	X	X
[13]	Securing training	✓	✓	X
[18]	accountability and fairness in training	✓	X	X
[21]	Consensus-based model aggregation	✓	✓	X
Our works	Secure Ecosystem	✓	✓	✓

**Table 2:** Training performance analysis.

Paper	Dataset	Baseline	Train Accuracy
[20]	MNIST	96.7%	97%
[20]	MNIST	90%	85%
[13]	MNIST	-	92%
[18]	COVID-19 Chestxray	86.80%	89.09%
[21]	FEMNIST	90.20%	90.02%
Our Works	Stanford Cars	98.8%	99%

**Table 3:** Various execution time.

Operations	Time (ms)
Compound key Generation	6–10
Block creation (Avg)	3.37
Local Model Update	46
Local Training in a Single LN (avg)	5280
Blockchain Write for Gradient	360
Aggregation (including consensus)	3000–5000
BC write for Gradient Average	261
Global Model Update	79

## Data Availability

Not applicable.
